# Enhanced temporal encoding-decoding for survival analysis of multimodal clinical data in smart healthcare

**DOI:** 10.1186/s42492-025-00209-7

**Published:** 2025-12-12

**Authors:** Xiaofeng Zhang, Zijie Pan, Yuhang Tian, Lili Wang, Tingting Xu, Li Chen, Xiangyun Liao, Tianyu Jiang

**Affiliations:** 1 College of Artificial Intelligence, Nankai University, Tianjin 300350, China; 2 Shenzhen Institute of Advanced Technology, Chinese Academy of Sciences, Shenzhen 518055, Guangdong China; 3 Emergency Department, the Ninth Medical Center of Chinese PLA General Hospital, Beijing 100101, China; 4 General Medicine Department, the First Medical Center of the PLA General Hospital, Beijing 100039, China; 5 Guangdong Provincial Key Laboratory of Computer Vision and Virtual Reality Technology, Shenzhen Institutes of Advanced Technology, Chinese Academy of Sciences, Shenzhen 518055, Guangdong China

**Keywords:** Digital health, Smart healthcare system, Facial feature detection, Encoding-decoding, Multimodal clinical data

## Abstract

Effective survival analysis is essential for identifying optimal preventive treatments within smart healthcare systems and leveraging digital health advancements; however, existing prediction models face limitations, primarily relying on ensemble classification techniques with suboptimal performance in both target detection and predictive accuracy. To address these gaps, this paper proposes a multimodal framework that integrates enhanced facial feature detection and temporal predictive modeling. For facial feature extraction, this study developed a lightweight face-region convolutional neural network (FRegNet) specialized in detecting key facial components, such as eyes and lips in clinical patients that incorporates a residual backbone (Rstem) to enhance feature representation and a facial path aggregated feature pyramid network for multi-resolution feature fusion; comparative experiments reveal that FRegNet outperforms state-of-the-art target detection algorithms, achieving average precision (AP) of 0.922, average recall of 0.933, mean average precision (mAP) of 0.987, and precision of 0.98–significantly surpassing other mask region-based convolutional neural networks (RCNN) variants, such as mask RCNN-ResNeXt with AP of 0.789 and mAP of 0.957. Based on the extracted facial features and clinical physiological indicators, this study proposes an enhanced temporal encoding-decoding (ETED) model that integrates an adaptive attention mechanism and a gated weighting mechanism to improve predictive performance, with comparative results demonstrating that the ETED variant incorporating facial features (ETEncoding-Decoding-Face) outperforms traditional models, achieving an accuracy of 0.916, precision of 0.850, recall of 0.895, F1 of 0.884, and area under the curve (AUC) of 0.947–outperforming gradient boosting with an accuracy of 0.922, but AUC of 0.669, and other classifiers in comprehensive metrics. The results confirm that the multimodal dataset (facial features + physiological indicators) significantly enhances the prediction accuracy of the seven-day survival conditions of patients. Correlation analysis reveals that chronic health evaluation and mean arterial pressure are positively correlated with survival, while temperature, Glasgow Coma Scale, and fibrinogen are negatively correlated.

## Introduction

### Survival analysis in chronic progressive diseases: challenges and the rationale for multimodal data-integrated survival prediction

Survival condition analysis is fundamental in medical and biological research and provide critical insights into disease progression [1], therapeutic efficacy [2], and treatment planning [3]. In clinical practice, predicting survival outcomes for patients with chronic progressive diseases, such as hepatocellular carcinoma and heart failure remains challenging. Clinicians often face two intertwined practical dilemmas. First, disease progression manifests through both systemic physiological changes (e.g., abnormal liver function indices and fluctuating heart rates) and overt physical signs (e.g., scleral jaundice and facial edema), and integrating these heterogeneous data–structured lab results and unstructured facial features–into a unified assessment is clinically cumbersome. Furthermore, fragmented evaluations, such as relying solely on blood tests or visual observation, frequently lead to delayed detection of disease deterioration [4]. Second, the importance of clinical features varies dynamically across disease stages, for example, in early heart failure, vital signs may be more predictive, whereas in advanced stages, liver function indices become critical. Traditional static models, such as fixed-weight Cox models, fail to adapt to such temporal variations, resulting in inaccurate long-term survival estimates and suboptimal treatment decisions [5].

These practical challenges highlight the need for a novel framework that can synergistically leverage multimodal data, while adapting to temporal variations in patient conditions. This study specifically addressed the problem of survival prediction in patients with chronic progressive diseases using two-year longitudinal data, focusing on predicting 12-month and 24-month survival outcomes by integrating three core data modalities: routine blood test indices, vital signs, and facial imaging data. Unlike previous studies that focused solely on clinical features or isolated facial analysis, this work establishes a unified analytical pipeline to model the interplay between the systemic physiological status and facial manifestations of disease progression.

**Fig. 1:**
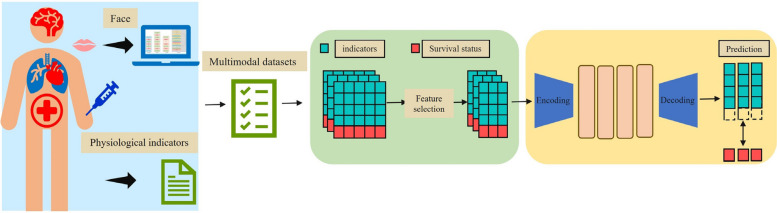
Algorithm framework from clinical medical treatment to intelligent health

Contemporary methodologies that integrate clinical physiological analysis with facial feature recognition substantially enhance treatment planning by utilizing of comprehensive multimodal datasets that encompass both physiological and facial characteristics to predict patient survival outcomes at specified temporal intervals. This integrated approach transcends conventional methodologies that rely exclusively on clinical features and medical histories, which frequently yield incomplete assessments of disease conditions and survival probabilities. The synergistic integration of facial feature analysis and physiological monitoring facilitates precise and comprehensive survival predictions [6], enabling clinicians to obtain a holistic understanding of patient conditions and prognostic outcomes, as illustrated in Fig. 1.

Facial feature recognition facilitates insights into patient health status through systematic analysis of facial attributes [7, 8]. These facial characteristics are valuable indicators of physiological conditions [9]. Through a comprehensive analysis of facial components, it is feasible to predict survival outcomes and disease progression [10]. Physiological indicators represent the functional state of the body quantitatively and are essential variables in survival analysis models [11]. The implementation of neural network methodologies enables efficient analysis of extensive clinical datasets, revealing correlations between physiological indicators and survival durations. The development of survival analysis models incorporating both clinical physiological indicators and facial feature recognition enhances the precision and reliability of survival predictions [12, 13]. These models facilitate evidence-based healthcare decision making by providing a more robust methodology for predicting patient survival outcomes.

### Related work in disease detection and survival prediction

Recently, several researchers have focused on predicting and analyzing the mortality caused by various diseases, resulting in the development of a range of effective methodological frameworks. Survival analysis plays a crucial role in the evaluation of gliomas. Wu et al. [14] proposed a multi task learning approach that integrates survival analysis with semi-supervised tumor segmentation. The creation of personalized dynamic risk profiles is essential for customizing disease management strategies. Sun et al. [15] developed a multi layer neural network survival model for precise survival analysis. Computer vision and neural networks are commonly used to diagnose dental diseases using X-ray imaging. Fatima et al. [16] introduced a lightweight mask RCNN model for disease detection. Classification techniques have been widely used in cancer survival analyses to predict patient survival and estimate life expectancy [17]. Deep learning technology has facilitated the identification of diseases from raw facial images, Jin et al. [18] employed deep transfer learning for computer-aided facial diagnosis.

With the growing emphasis on health, research on disease survival prediction has increased. Zhao et al. [19] proposed the use of the mask R-CNN algorithm to segment mandibular neural canals in dental X-rays. Hoorali et al. [20] introduced a modified mask RCNN system as an automated diagnostic tool for anthrax tissue samples. Indumathi and Siva [21] proposed a hybrid framework that combines a bi-directional memory network with a mask region-based network for lung disease prediction. Fan et al. [22] presented a computer-aided diagnostic system utilizing a 3D-mask region convolutional neural network. To assess fetal growth, mask R2CNN, a fully automated deep learning-based method, was introduced for fetal head circumference measurement from ultrasound images [23]. Artificial intelligence-assisted cervical cytology was developed for oral cytology [24]. Evain et al. [25] proposed a method that uses a mask RCNN to determine the benign or malignant nature of breast nodules from initially labeled 2D ultrasound data. Long et al. [26] proposed a flexible probabilistic mask R-CNN. Hussain et al. [27] introduced a segmentation framework based on the U-Net architecture to diagnose pre cancerous and cancerous lesions of the cervix using Pap smear analysis. Vieira et al. [28] proposed a multi pathology system utilizing an improved mask RCNN for multi object detection in WCE images. Mask RCNN architecture was used for segmentation in osteoarthritis, accommodating different tissue scales and magnetic resonance imaging sequences [29]. Masood et al. [30] trained a mask region-based CNN through transfer learning for accurate brain tumor segmentation and classification. Jin et al. [31] developed a CNN-based automatic endoscopic detection system for early gastric cancer using mask regions. A deep learning model for facial photo-based detection was developed and validated to investigate the correlation between facial features and coronary artery disease risk [32]. The study improves facial weakness detection accuracy by combining directed gradient histogram features from a single image [33].

Wu et al. [34] proposed a two-stage survival prediction model called, ICSPM. Wang et al. [35] introduced the GCGCN, a cancer survival prediction method for cancers with distinct clinical outcome variations. Chen et al. [36] proposed an enhanced survival prediction model using self-supervised learning to evaluate tumor treatment choices. Ma et al. [37] introduced XGBLC, an enhanced survival prediction model based on XGBoost, for survival analysis based on gene expression profiling. Li et al. [38] developed an ANN-based survival prediction model for patients. Lee et al. [39] identified genomic biomarkers and developed a computational survival prediction model for colon cancer. Pongnikorn et al. [40] developed a new survival prediction model for breast cancer risk using modern statistical methods. Atallah et al. [41] developed a data mining method to predict five-year graft survival in kidney transplantation immunosuppressive therapy. Ding et al. [42] improved cervical cancer treatment using an miRNA-based machine learning model for accurate classification prediction. Hao et al. [43] proposed a multi omics joint-learning method using whole-genome data to accurately predict the expected survival of patients with cancer. Wiltgen et al. [44] investigated survival prediction in glioblastoma patients using quantitative image analysis and radiomic features after radiation treatment. 

### Survival prediction solutions based on enhanced temporal encoding-decoding framework and multimodal data integration

The conventional RegNet architecture has a limited in performance owing to its single-stem design, which creates sampling bottlenecks and reduces its adaptability to multi-scale medical imaging data. To address these limitations, a residual stem (Rstem) module that enhances network depth and significantly improves feature extraction capabilities was introduced. This residual architecture effectively mitigates feature information loss during processing, which is an innovation distinct from recent stem modifications in ResNet variants that primarily focus on width adjustment rather than depth optimization. For feature refinement and output generation, this study implemented a face pyramid aggregation feature pyramid network (FPAFPN) to enhance both the local feature sensitivity and global semantic understanding. Unlike the standard FPN, which uses fixed upsampling ratios, the FPAFPN’s triple upsampling strategy with adaptive scaling factors enables the precise alignment of facial micro-features (e.g., scleral jaundice) with macro-structural changes (e.g., facial edema), which are critical for early disease detection.

In medical data classification and prediction, traditional architectures struggle to handle high-dimensional features and variable-length sequences. The enhanced temporal encoding-decoding (ETED) framework addresses these issues through the integration of gated mechanisms and adaptive weighting, enabling the effective processing of tabular data while enhancing key feature identification and long-range dependency modeling. Unlike transformer-based models with uniform temporal attention, ETED’s dynamic gating adjusts feature weights based on clinical event triggers (e.g., medication changes and spikes in lab results), achieving context-aware temporal modeling and significantly improving the handling of high-dimensional non-sequential data as well as predictive classification performance. This study makes three contributions: first, it resolves the limitations of traditional architectures via a gated mechanism with adaptive weighting, enabling context-aware modeling of clinical events–outperforming transformer models reliant on uniform temporal attention; second, empirical results show that integrating high-precision facial feature detection (outperforming state-of-the-art mask RCNN variants, Table 1) with temporal encoding-decoding enhances predictive performance, providing a novel multimodal framework for medical data processing; third, ETED demonstrates superior performance across key metrics (e.g., area under the curve (AUC) of 0.947 for ETEncoding-Decoding-Face *vs* 0.739 for XGBC, Table 2), validating its practical value for reliable clinical prediction and setting a new benchmark in multimodal medical data classification.

**Table 1:** Comparison of results of six algorithms for target detection

Algorithm	AP	AR	mAP	Precision
Mask RCNN-ResNet	0.703	0.732	0.909	0.96
Mask RCNN-ResNeSt	0.465	0.739	0.896	0.62
Mask RCNN-ResNeXVd	0.700	0.722	0.936	0.96
Mask RCNN-ResNeXt	0.789	0.859	0.957	0.92
Mask RCNN-RegNet	0.697	0.736	0.955	0.95
Ours	0.922	0.933	0.987	0.98

*AP* Average precision, *AR* Average recall, *mAP* Mean average precision

The methods proposed for eye and lip target detection for clinical patients outperform other target detection algorithms

**Table 2:** Performance comparison of ETED model against traditional models

Algorithm	Accuracy	Precision	Recall	F1-score	AUC
Gaussian NB Classifier	0.711	0.464	0.376	0.416	0.339
MLP Classifierc	0.889	0.547	0.565	0.555	0.461
Logistic Regression Classifier	0.789	0.467	0.418	0.441	0.471
Decision Tree Classifier	0.867	0.530	0.553	0.535	0.552
XGBC Classifier	0.878	0.612	0.747	0.643	0.739
K Neighbor Classifier	0.622	0.482	0.424	0.410	0.449
Gradient Boosting Classifier	0.922	0.649	0.676	0.661	0.669
Random Forest Classifier	0.867	0.470	0.459	0.464	0.701
AdaBoost Classifier	0.878	0.470	0.465	0.467	0.631
ETEncoding-Decoding	0.887	0.845	0.926	0.845	0.845
ETEncoding-Decoding-Face	0.916	0.850	0.895	0.884	0.947

The main contributions of this research are as follows:

1. This study developed an ETED framework to predict patient survival status from multimodal clinical data. This novel approach demonstrates superior predictive accuracy compared with traditional ensemble machine learning models in survival analysis by incorporating clinical event-aware gating mechanisms that outperform time-invariant attention models in handling irregularly sampled medical data.
2. This study introduces an advanced face RCNN architecture that integrates RegNet with a multi-scale feature pyramid. The innovative lightweight Rstem design effectively mitigates gradient vanishing issues–a critical improvement over RegNetX which exhibits performance degradation in low-resolution facial imaging and the proposed FPAFPN mechanism significantly enhances both feature resolution and nonlinear representation capability.
3. The temporal prediction model incorporates an adaptive gated weighting mechanism that dynamically adjusts the feature importance across different temporal dimensions and hidden states, thereby enabling a more precise survival probability estimation. This mechanism specifically addresses the limitations of static feature weighting in Cox proportional hazards models when applied to patients with fluctuating clinical conditions.

## Methods

### ETED model for survival analysis on multimodal clinical data

This study proposes an ETED model for the survival analysis of multimodal clinical data, aiming to predict the survival status of clinical patients over a seven-day period. This dataset incorporates both facial features and clinical physiological indicators of patients. This study introduces the face RCNN algorithm for extracting facial features and utilizes the ETED model as a predictive classification framework for the multimodal clinical data.

This study proposes the use of the face RCNN algorithm as a recognition method for detecting facial feature targets. FRegNet is a lightweight network architecture, was proposed to optimized feature extraction efficiently. FRegNet incorporates the Rstem residual structure and address challenges such as gradient vanishing, slow convergence and overfitting. This design enhances the stability and performance of the network during feature extraction. This study proposes FPAFPN, a feature pyramid attention fusion network, for the output of feature information. FPAFPN integrates convolutional layers with double and triple up-sampling to enhance the nonlinear transformation, improve the feature resolution, and effectively capture detailed information. This integration significantly improves the overall performance of feature extraction and facilitates the detection of fine-grained facial features. In addition, this study proposes ETED, a novel approach for the predictive classification of tabular data. It incorporates an adaptive attention mechanism and gated weighting mechanism, effectively handling long-term dependencies and improving the classification performance. This approach leverages fully connected networks, activation functions, and long short-term memory (LSTM) to capture critical information and enhance the classification accuracy of tabular data.

### Face region convolutional neural network with residual RegNet and multi scale sampling pyramid

**Fig. 2:**
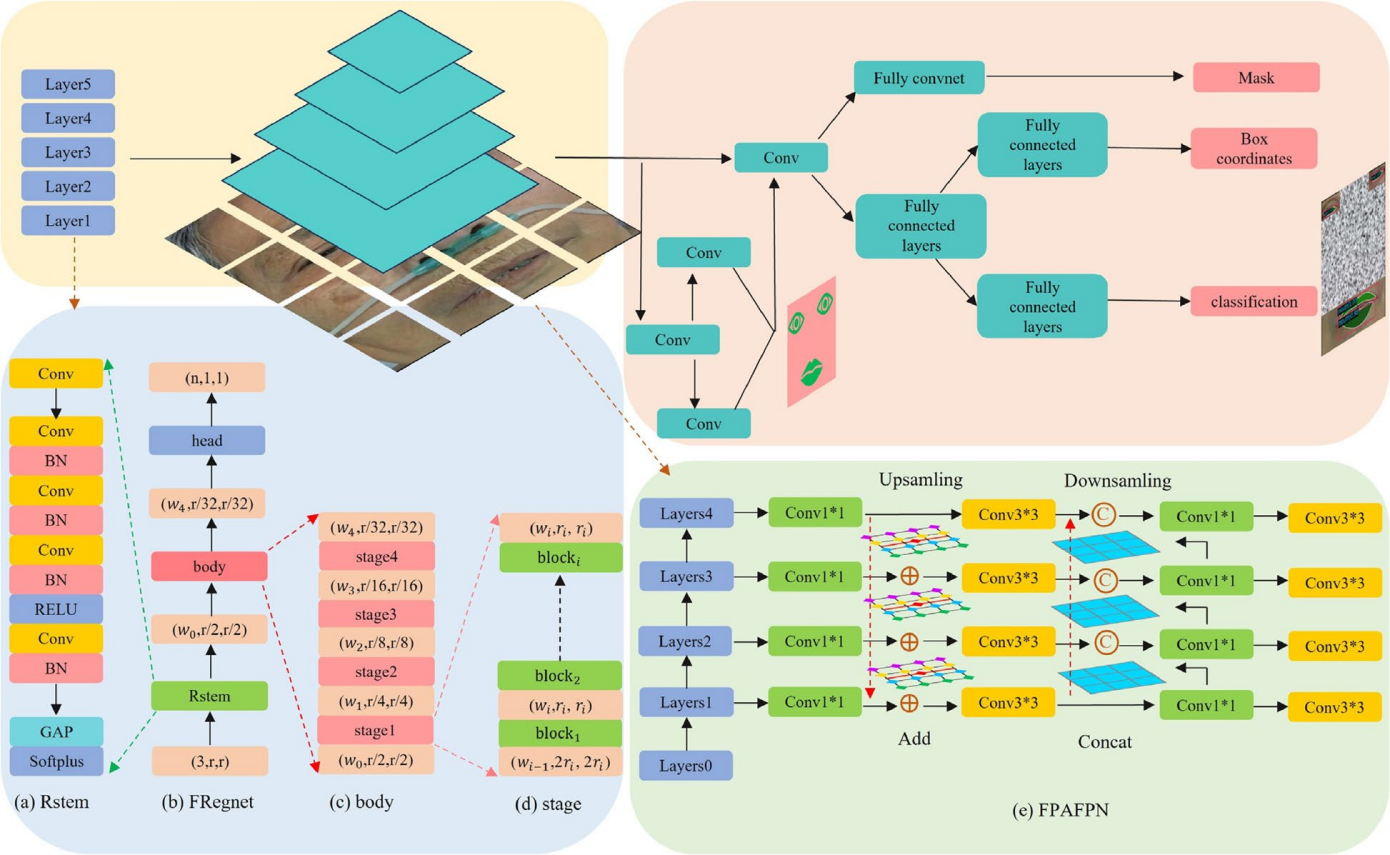
Face region convolutional neural network with residual RegNet and multi-scale sampling pyramid

For the feature extraction phase, this study proposes FRegNet, which integrates a stem with a deep residual function as its initial component, as shown in Fig. 2. The deep residual structure facilitates the direct transmission of feature information within the network, thereby mitigating information loss caused by multiple nonlinear transformations. Within the Rstem structure, the input information is divided into two pathways. One pathway delivers information to the output through cross-level connections, whereas the other undergoes further feature extraction operations before being added to the output. Global average pooling is employed to reduce the output of the global feature vector. Softplus, a smooth nonlinear activation function, was utilized in place of ReLU to address the gradient vanishing issue while reducing the number of parameters and computational complexity. The combination of global average pooling and SoftPlus not only accelerates the computation but also enhances the model’s feature representation capacity and overall performance.

This study proposes the use of the FPAFPN module as an up-sampling output component for feature information. The inclusion of a convolutional layer within the FPAFPN module facilitated nonlinear transformation and feature extraction. The fusion of features at multiple levels within the feature pyramid improved the generalization capability of the model. The double and triple up-sampling structures progressively restores the resolution of the feature map, aiding in the detection of small targets and capture of fine-grained details. Reducing the feature map size also reduces the complexity of the model. By combining low and high-level semantic information, FPAFPN enhances the transfer of semantic information and improves the model’s ability to integrate both detailed and abstract features, thereby enhancing the overall performance.

The formula for the deep residual mapping structure contained in the Rstem structure is as follows:

1 $${G}_{i}\left(x\right)=Relu\left(\sum\limits_{i=1}^{N}\left({k}_{ci}{x}_{i}+ {b}_{i}\right)\right)$$

2 $${G}_{\left\{i\right\}\left(x\right)}=Softplus\left(\left(\sum\limits_{i=1}^{N}{G}_{\left(i\right)}\left(x\right)+{k}_{di}{x}_{i}\right){k}_{GAPi}\right)$$

where $G$ is the output result of the backbone of the deep residual structure, $Q$ is the output result of the entire residual structure, $Relu$ and $Softplus$ are the activation functions, $K$ is the convolution operation of the convolutional layer, *b* is the bias result, ${k}_{GAP}$ is the global average pooling operation, and $x$ is the information of the input features.

The calculation process of FPAFPN mainly consists of two steps: bottom-up path aggregation and top-down feature aggregation, the specific formulas of which are shown below.

3 $${P}_{i}= Con\left({C}_{i}\right)+ H\left({P}_{i+1}\right)$$

4 $${F}_{i}= Con\left({P}_{i}\right)+ Con\left({F}_{i+1}\right)$$

5 $$H\left(x,y\right)=\sum\limits_{i}^{3}\sum\limits_{j}^{3}{Q}_{\left(i,j\right)}\delta \left({y}-{y}_{j}\right)\delta \left({x}-{x}_{i}\right)$$

where $P$ is the feature map of layer $i$, $C$ is the bottom-up feature map of layer $i$, $H$ is the double-three upsampling operation, $Con$ is the convolution operation, $F$ is the fused feature map of layer $i$, ${Q}_{(i,j)}$ is the input information, and ${\delta }_{i}(x)$ and ${\delta }_{j}(y)$ are the interpolation weighting factors in the horizontal and vertical directions.

### ETED

**Fig. 3:**
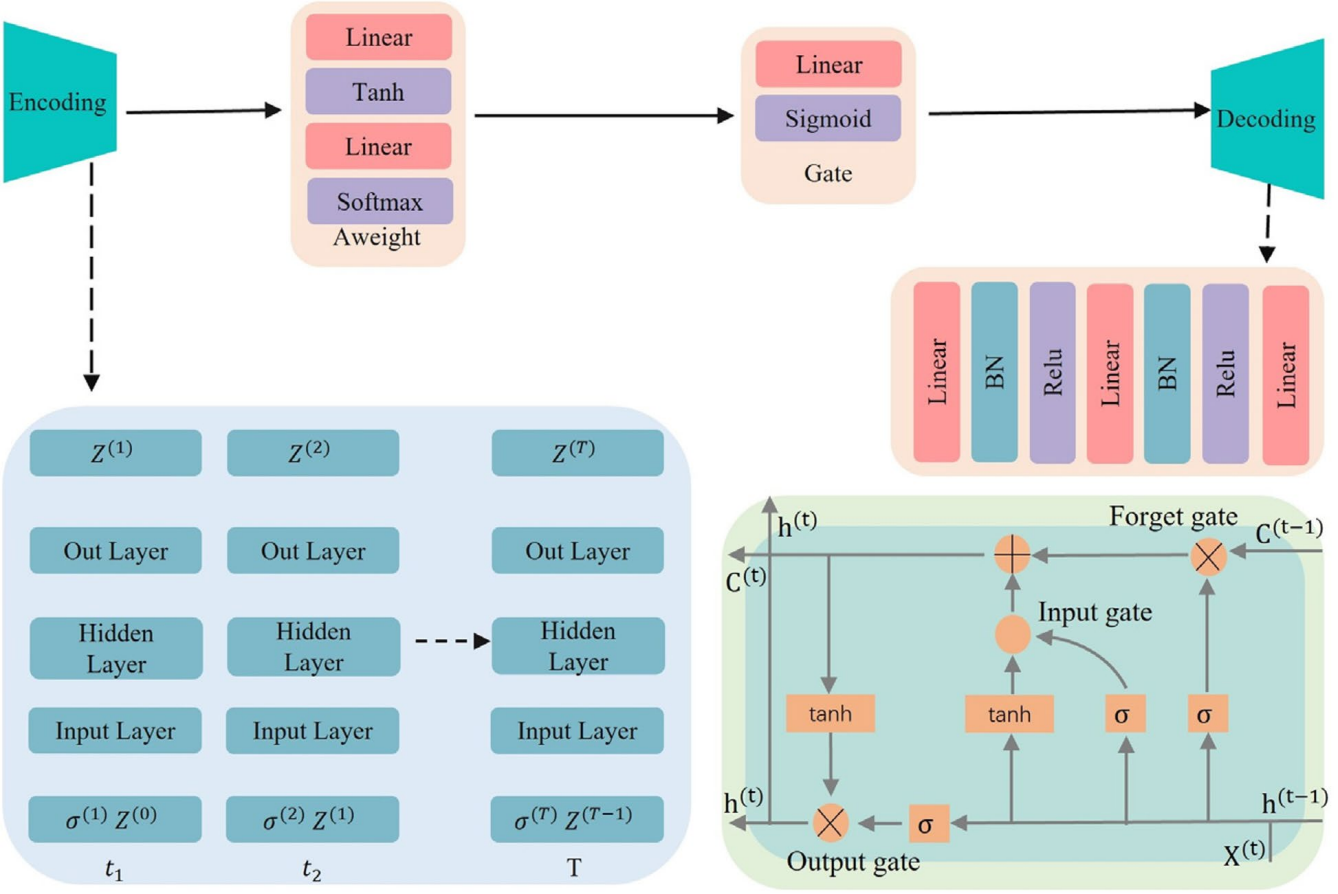
ETED

This study proposes an ETED module for constructing a predictive classification model for tabular data, as shown in Fig. 3. It integrates LSTM, a gated mechanism, an adaptive weighting mechanism, and a residual structure. LSTM is employed to process sequential data by utilizing the input, forget, and output gates. The gated mechanism modulates the output-state weights using sigmoid activation functions. The adaptive weighting mechanism leverages an attention layer to compute the weights for each time step and generates weighted outputs. The residual structure combines the weighted output with the original output through a residual connection to produce a predicted output. The ETED module effectively integrates these components to process tabular data and generate accurate predictive classification.

The LSTM unit consists of three primary components. The forget gate determines the information from the previous state that should be retained. The input gate regulates the weight of each input. The candidate state vector is computed by applying a nonlinear transformation to the current inputs. The current state is updated by combining the candidate state vector with the input gate output. The output gate controls the output information, and a new output vector is computed through a nonlinear transformation of the current state. The final output value is obtained by weighting and summing the output of the output gate and the new output vector. The internal structure of the LSTM unit is illustrated in the following diagram.

The computational process of LSTM consists of four steps: forget gate, input gate, memory cell and output gate. The specific calculation formula is as follows:

6 $${f}^{t}= sigmoid\left({W}_{f}{h}^{t-1}+{U}_{f}\cdot {\mathbb{Z}}^{i\times j}+ {b}_{f}\right)$$

7 $${i}^{t}= sigmoid\left({W}_{i}{h}^{t-1}+{U}_{i}{x}^{t}+ {b}_{i}\right)$$

8 $$C^t=f^tc^{t-1}+i^t\tanh\left(W_ch^{t-1}+U_cx^t+b_c\right)$$

9 $${o}^{t}= sigmoid\left({W}_{o}{h}^{t-1}+{U}_{o}{x}^{t}+ {b}_{o}\right)$$

10 $${h}^{t}= {o}^{t}\mathrm{tanh}({C}^{t})$$

where ${f}^{t}$, ${i}^{t}$, and ${o}^{t}$ are the activation values of the forget input and output gates, respectively; ${C}^{t}$ is the current cell state; ${h}^{t}$ is the current hidden state; ${\mathbb{Z}}$ is the set of clinical characteristics of the patient; $W$, $U$, and $b$ are the weights and biases; and, $sigmoid$ and $tanh$ are the activation functions.

11 $$f\left(z_{ij}^{\left(t\right)}\right)=\begin{bmatrix}z_{11}^t&\cdots&z_{1j}^t\\\vdots&\ddots&\vdots\\z_{i1}^t&\cdots&z_{ij}^t\end{bmatrix}=\begin{bmatrix}x_{11}^t&\cdots&x_{1j}^t\\\vdots&\ddots&\vdots\\x_{i1}^t&\cdots&x_{ij}^t\end{bmatrix}\begin{bmatrix}w_{11}^t&\cdots&w_{1j}^t\\\vdots&\ddots&\vdots\\w_{i1}^t&\cdots&w_{ij}^t\end{bmatrix}+\begin{bmatrix}\alpha_{11}^t&\cdots&\alpha_{1j}^t\\\vdots&\ddots&\vdots\\\alpha_{i1}^t&\cdots&\alpha_{ij}^t\end{bmatrix}$$

12 $$weight=softmax\left(\mathrm{tanh}\left(f\left(z_{\left\{ij\right\}}^{\left\{\left(t\right)\right\}}\right)\right)\right)$$

13 $$R = \sum\limits_{i=1}^{t}\left(Aweight\cdot {\mathbb{Z}}^{i\times j}\right)$$

14 $$Gate = sigmoid\left( R \cdot f\left({z}_{i,j}^{t}\right)\right)$$

15 $${F}_{i}\left(x\right)= BN\left(Relu\left(f\left( {\mathbb{Z}}^{i\times j}\right)\right)\right)+ {B}_{i}$$

where $z$ is the output that passes through the layer of the network, $(i,j)$ is the output data, $i$ represents the sample classification,$j$ represents the sample features, $t$ is the layer of the network, $x$ is the input to the network, $w$ is the weight coefficient of the layer of the network, and $\alpha$ is the bias coefficient of the layer of the network. ${\mathbb{Z}}$ is the set of clinical characteristics of the patient,$Aweight$ is the adaptive weighting factor, $Gate$ is the output of the gating mechanism, and $B$ is the bias factor. $Relu$, $softmax$, $sigmoid$ and $tanh$ are the activation function.

## Results

### Acquisition of facial features based on face RCNN

The clinical dataset used in this study was collected from the Emergency Department of the PLA Support Force Characteristic Medical Center and the General Medicine Department of the First Medical Center of the PLA General Hospital. It comprises 298 samples, all of which are associated with clinical physiological indicators. Facial features were extracted using face-RCNN, with a focus on the variability of patient eye movements. These facial features, in conjunction with the clinical physiological indicators, were integrated to form a multimodal dataset. The ETED network was subsequently employed for the prediction and classification tasks. The detailed explanation of the physiological indicators involved in this paper is shown in Appendix A.

In this study, facial information of 298 patients was used for eye target detection. Five other target detection algorithms were compared with the proposed method. The results of the six algorithms are listed in Table 1. The proposed method demonstrated superior accuracy in detecting the eyes of clinical patients compared to other algorithms.

**Fig. 4:**
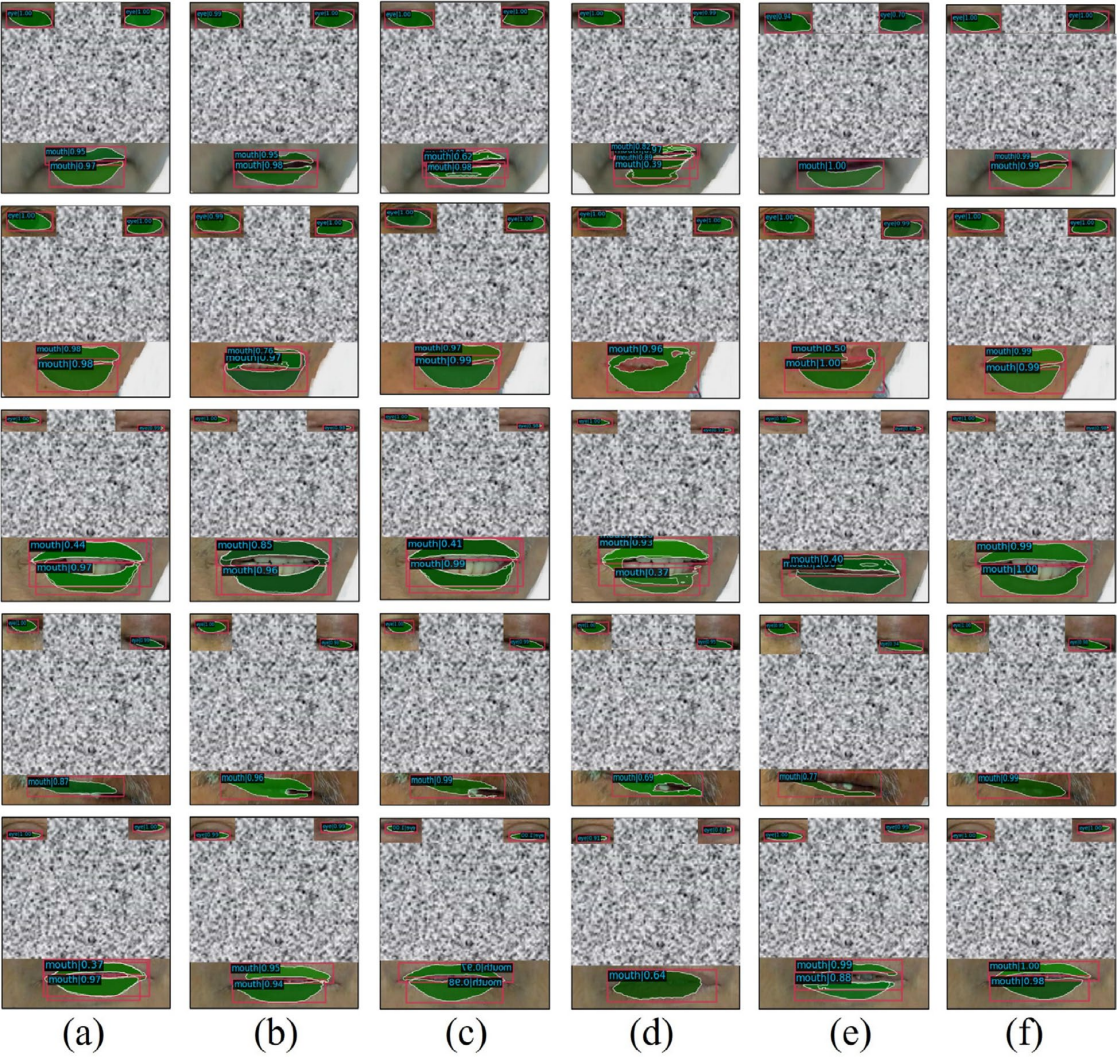
Comparison of the detection results of the six target detection algorithms, with parts of the face obscured to protect patient privacy, (**a**) is Mask RCNN-ResNet, (**b**) is Mask RCNN-ResNetVD, (**c**) is Mask RCNN-ResNeXt, (**d**) is Mask RCNN-ResNest, (**e**) is Mask RCNN-Regnet, and (**f**) is Ours

Figure 4 illustrates the results of the six target detection algorithms for mouth detection in facial images. The small boxes indicate the category of the detected object, and the number within each box represents the confidence level of detection. The confidence reflects the certainty of the detection results, with higher values indicating greater confidence. As shown in Fig. 4, the proposed algorithm outperforms the other five algorithms. The other algorithms exhibited lower detection accuracy, unclear target detection regions, and instances of overlapping or undetected anchor boxes. Consequently, the algorithm proposed in this study demonstrated superior detection accuracy compared with the other algorithms.

**Fig. 5:**
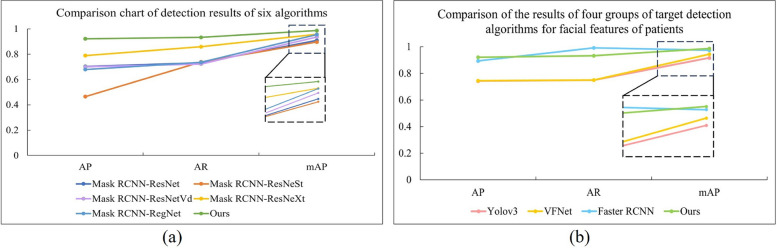
Comparison of algorithms. **a** Comparison of detection results of six target feature extraction algorithms; **b** Comparison of results of four target detection algorithms for patient facial feature extraction

AP, AR, and mAP were used as evaluation metrics for the object detection tasks. AP is computed by assessing the accuracy of each category across various intersection-over-union (IoU) thresholds. A higher AP value signifies enhanced detection precision. AR is determined by averaging the recall of each category at different IoU thresholds. A higher AR value indicates an improved model recall. mAP is derived by averaging the AP values of all categories across multiple IoU thresholds. A higher mAP value reflects superior overall detection precision of the model. Figure 5 compares target detection performance via two subfigures: (a) assesses six algorithms (five mask R-CNN variants and “Ours”) for general target feature extraction, while (b) evaluates four methods (Yolov3, VFNet, Faster R-CNN, and “Ours”) for patient facial feature detection, using AP, AR, and mAP. In both experiments, “Ours” outperformed the baseline algorithms, demonstrating superior efficacy in both general and specialized detection tasks.

### Predictive classification based on ETED

In this study, a predictive classification model was developed using physiological indices related to the survival status of patients. The objective of this study was to demonstrate its superior classification and prediction performance compared with other commonly used models in clinical survival analysis. To this end, a comparative experiment was conducted using nine predictive classification models. The results of the experiments are listed in Table 2. Evaluation of the predictive classification outcomes was based on several performance metrics, including accuracy, precision, recall, F1-score, and AUC.

As presented in Table 2, the proposed survival condition prediction model outperformed all other models in terms of predictive classification accuracy.

**Fig. 6:**
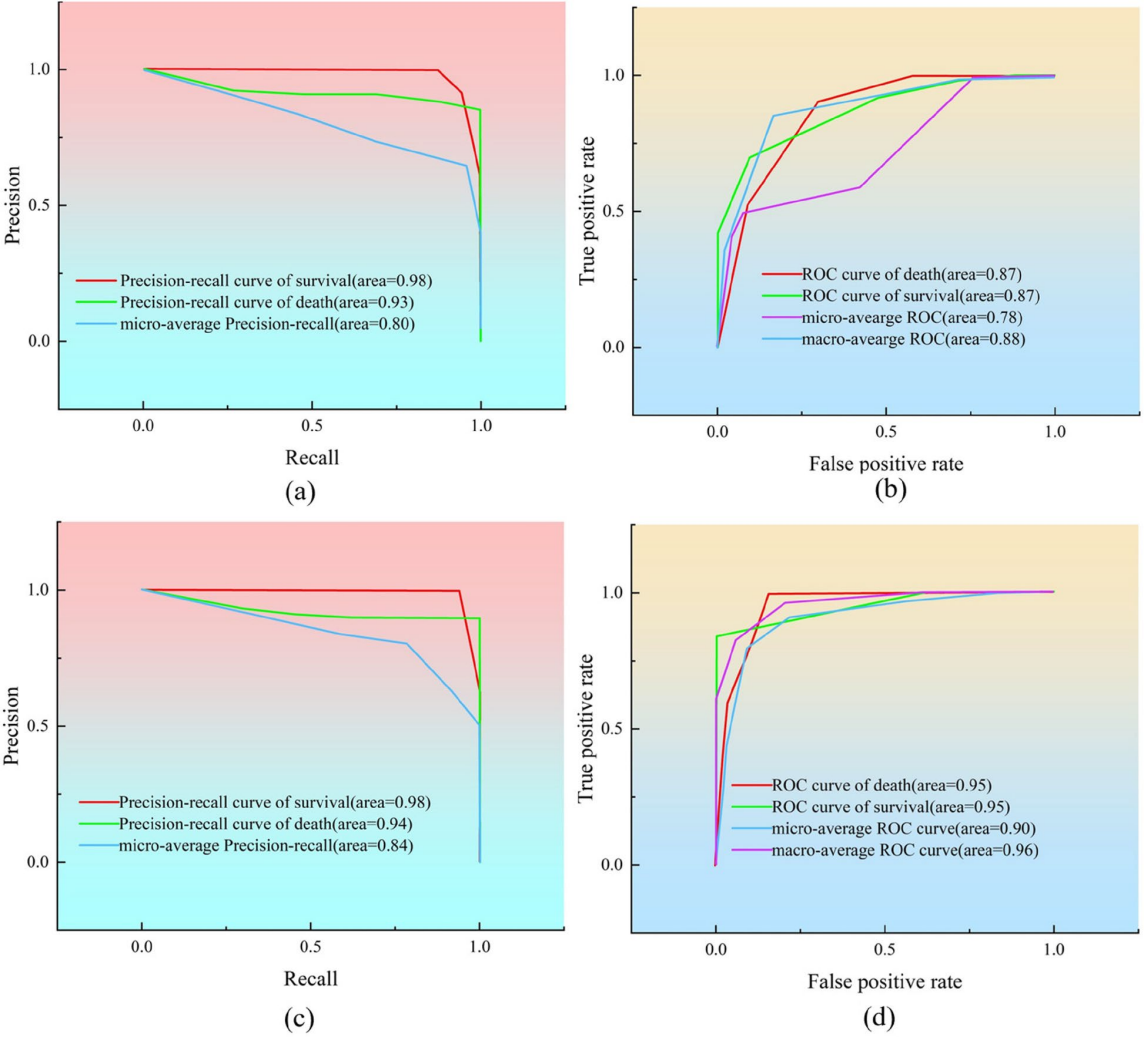
(**a**) and (**b**) show the single classification prediction of the ETED model for the clinical physiology dataset, and (**c**) and (**d**) show the results of the multimodal prediction of the ETED model by combining the clinical physiology dataset and extracted facial features

From Fig. 6, it is evident that the integration of multimodal clinical data, combining facial feature data with clinical physiological indicators, results a higher accuracy in predicting survival status compared to using clinical physiological data alone. The precision-recall curve illustrates the relationship between precision and recall based on the classifier’s prediction results, whereas the receiver operating characteristic curve depicts the true and false positive rates relative to the prediction outcomes.

### Survival analysis based on multimodal clinical data

In the subsection on categorical prediction of medical tabular data, 300 medical tabular samples were utilized, comprising 36 clinical physiological indicators and one categorical label. Appendix A presents the indicators. The categorical label consists of two classes: the first indicates that the clinical patient will die within seven days, and the second indicates that the clinical patient will survive beyond seven days.

Table 3 presents the results of the analysis of clinical physiological indicators associated with survival status over a 7-day period, highlighting the significant correlations (*P* < 0.05) between the following indicators: temperature, systolic pressure, MAP, respiratory rate, Na, Glasgow Coma Scale (GCS), chronic health evaluation, BE, AST, BIL, PT, APTT, and fibrinogen (FIB). These findings suggest a relationship between these physiological indicators and patient survival during the seven-day period. To further investigate the clinical and physiological characteristics related to survival status, binary logistic regression analysis was performed. The results, as presented in Table 4, combine the outcomes of both the correlation and binary logistic regression analyses. This integrated analysis offers deeper insights into the clinical physiological indicators associated with survival within the a seven-day period.

**Table 3:** Correlation analysis of clinical physiological characteristics related to survival

Variable	Seven-day death data of patients	Seven-day survival data of patients	*P* value
Gender (male)	8	179	
Age	66.21	63.97	
Apache	17.50	11.93	0.307
Temperature	37.07	36.85	0.021
Systolic pressure	100.64	126.78	0.001
Diastolic pressure	59.36	71.86	0.100
MAP	70.14	90.17	< 0.001
Heart rate	119	91.62	0.896
Respiratory rate	22.29	19.90	0.010
Partial pressure of oxygen	87.57	107.94	0.653
pH	7.44	7.43	0.631
Na	137.46	136.31	0.026
K	4.34	7.37	0.665
Cr	177.44	179.92	0.309
HCT	28.84	31.10	0.720
WBC	14.36	11.14	0.122
GCS	2.86	0.70	< 0.001
AE score	4.07	3.71	0.743
Chronic health evaluation	0.36	0.92	0.013
Hemoglobin	97.14	103.91	0.228
Anemia	0.71	0.73	0.752
Platelet	193.79	202.45	0.359
PCO_2_	28.41	31.82	0.052
Lac	3.04	1.52	0.054
BE	-1.58	-0.72	0.025
ALT	58.88	46.83	0.737
AST	115.50	54.53	0.047
BUN	21.58	12.77	0.060
BSL	9.28	8.99	0.781
ALB	29.40	33.25	0.548
BIL	39.51	20.25	0.039
Serum calcium	2.06	2.08	0.876
Amylase	99.06	82.79	0.145
Lipase	41.88	81.81	0.254
PT	33.37	18.52	< 0.001
APTT	45.56	39.66	< 0.001
FIB	4.66	3.98	0.008
D DIMER	5.83	4.12	0.420

**Table 4:** Results of dichotomous logistic regression model analysis

Variable	B	SE	Beta	t	95%CI (Low)	95%CI (Up)	OR
Gender (male)	-0.004	0.026	-0.010	-0.163	-0.055	0.047	0.996
Age	0.002	0.002	0.125	0.707	-0.003	0.006	1.002
Apache	-0.006	0.004	-0.160	-1.564	-0.014	0.002	0.994
Temperature	-0.001	0.020	-0.004	-0.058	-0.040	0.038	0.999
Systolic pressure	0	0.001	0.046	0.363	-0.001	0.002	1
Diastolic pressure	-0.001	0.001	-0.084	-0.659	-0.004	0.002	0.999
MAP	0.001	0.001	0.137	0.886	-0.001	0.004	1.001
Heart rate	-0.001	0.001	-0.118	-1.812	-0.002	0	0.999
Respiratory rate	-0.003	0.004	-0.051	-0.838	-0.011	0.001	0.997
Partial pressure of oxygen	0	0	0.085	1.435	0	0.001	1
pH	0	0	0.014	0.250	-0.005	0.003	1
K	-0.001	0.002	-0.051	-0.605	-0.005	0.001	0.999
Na	0	0	0.014	0.215	-0.033	0.003	1
Cr	0	0	0.269	2.970	0	0	1
HCT	0	0	0.007	0.119	-0.003	0.004	1
GCS	0.006	0.007	-0.067	-0.850	-0.019	0.003	0.994
WBC	0.007	0.002	0.007	0.994	-0.003	0.003	1
AE score	0.003	0.018	-0.034	-0.192	-0.038	0.031	0.997
Chronichealth evaluation	0.025	0.007	0.225	3.342	0.010	0.040	1.025
Anemia	0.001	0.041	0.043	0.086	-0.044	0.126	1.042
Hemoglobin	0.041	0.043	0.178	1.593	0.003	0.003	1.001
Platelet	0	0	-0.028	-0.450	0	0	1
PCO_2_	0	0	-0.009	-0.156	-0.038	0.004	1
ALT	0	0	0.009	0.244	-0.003	0.007	1
BE	-0.020	0.002	-0.156	-2.339	-0.007	-0.003	0.980
Lac	0	0.004	-0.004	-0.003	-0.003	0.001	1
AST	0	0	-0.156	-1.582	-0.001	0	1
BSL	0.003	0.002	0.067	1.076	-0.008	0.008	1.003
BUN	-0.005	0.003	-0.290	-3.133	-0.002	-0.002	0.995
ALB	-0.002	0.002	-0.059	-0.834	-0.006	0.002	0.998
BIL	-0.001	0	-0.145	-2.397	-0.001	0	0.999
Serum calcium	-0.046	0.061	-0.048	-0.746	-0.167	0.075	0.955
Amylase	0	0	-0.055	-0.804	0	0	1
Lipase	0	0	0.172	2.412	0	0	1
PT	-0.002	0.001	-0.134	-1.983	0.003	0.003	0.998
APTT	0.002	0.001	0.019	0.003	0.003	0	1.003
FIB	-0.011	0.003	-0.099	-1.574	-0.025	-0.007	0.989
Constant	-0.011	1.886	-0.099	-0.724	-1.497	1.175	0.998

**Fig. 7:**
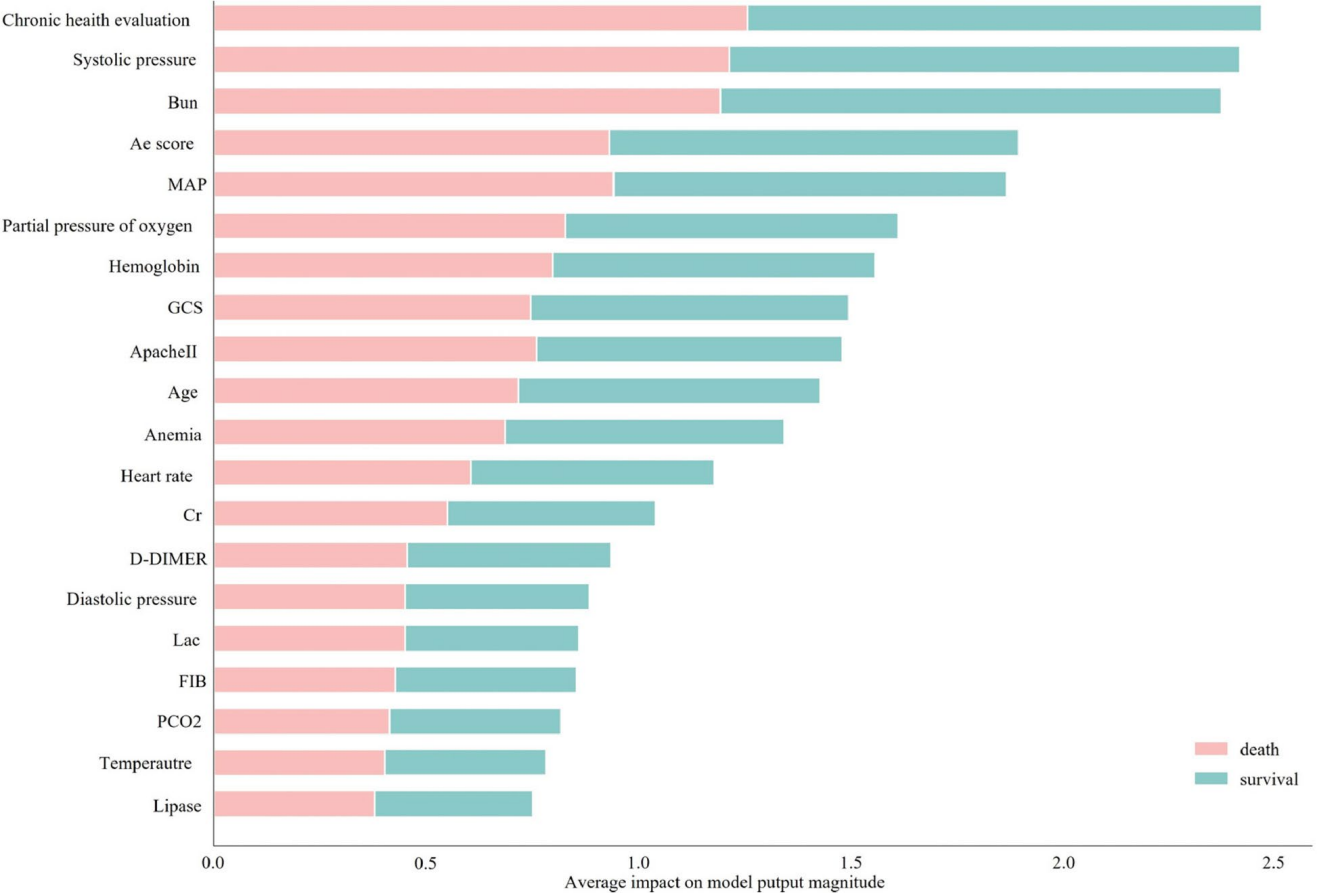
Average impact model output magnitude

Figure 7 illustrates the 20 physiological indicators with the greatest influence on the survival status. Correlation analysis and regression coefficients were used to select the relevant physiological indicators. A clinical prediction model for patient survival status within seven days was subsequently developed based on multimodal clinical data, integrating both physiological indicators and facial features.

After performing feature selection using the ETED model on clinical patient data, 10 clinical physiological indicators that significantly impacted survival status were identified. By applying the proposed ETED model to predict and classify multimodal clinical data, valuable insights were gained regarding the factors that influence survival status. The identified physiological indicators included the chronic health evaluation score, temperature, MAP, GCS, BIL, and FIB. Furthermore, the flexibility of eye movements, which is a facial feature, was found to have a significant effect on patient survival.

According to the experimental results, an increase of one unit in chronic health evaluation was associated with a 0.025 increase in the probability of survival. In contrast, a one-unit increase in temperature was linked to a 0.001 decrease in the probability of survival. Similarly, each one-unit increase in MAP was associated with a 0.001 increase in the probability of survival. Furthermore, a one-unit decrease in the GCS score corresponded to a 0.006 increase in the probability of survival. Additionally, for every unit increase in FIB, the probability of survival decreased by 0.011.

**Fig. 8:**
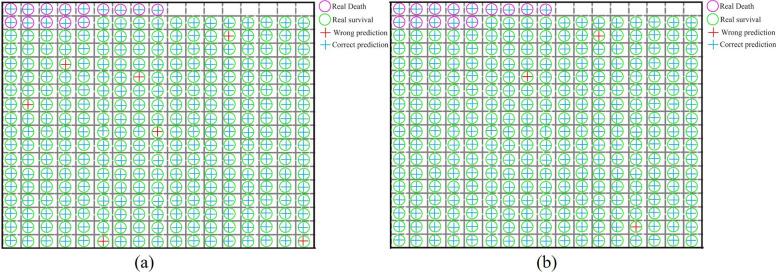
Comparison of seven-day survival prediction outcomes: clinical physiological indicators *vs* multimodal dataset. **a** Survival prediction within 7 days based on clinical physiological indicators; **b** Survival prediction within 7 days based on the multimodal dataset

This study predicted the survival status of 298 patients over a seven-day period. Survival was predicted based on clinical physiological indicators. As shown in Fig. 8(a), the number of deaths within seven days was accurately predicted, and there were seven prediction errors for the number of survivors. Subsequently, predictive analysis was performed on a multimodal dataset comprising both physiological indicators and facial data. As shown in Fig. 8(b), the number of deaths within seven days was correctly predicted, and the number of prediction errors for the seven-day survivors decreased to three. This demonstrated that the survival prediction framework proposed in this study significantly improves the prediction accuracy of the clinical multimodal dataset.

## Discussion

The integration of computer vision and predictive classification techniques in clinical medical applications has enabled the development of patients survival prediction models. This approach supports physicians in evaluating the disease severity and prognostic risk, thereby guiding treatment decisions. By extracting detailed features from medical images and enhancing the accuracy of survival prediction models, computer vision provides valuable clinical insights when integrated with biological and psychological factors, thereby contributing to a more holistic understanding of a patient’s overall health status. By combining diverse technologies and data sources, a more accurate and comprehensive survival prediction model can be developed to facilitate early detection of health risks and improve patient outcomes. The fusion of computer vision and predictive classification techniques is essential for to ehance the accuracy of patient survival predictions.

In this study, face RCNN was proposed to extract facial feature information, with a particular focus on the faces of patients. To address challenges such as slow convergence and overfitting, FRegNet, which is a lightweight network architecture, was proposed. The initial component of FRegNet uses a stem structure with a deep residual architecture. FPAFPN was proposed as an up-sampling structure that enhances the network’s ability to detect fine-grained details and model nonlinearity. Compared with traditional methods, the proposed target detection algorithms demonstrated higher evaluation accuracy. Notably, these algorithms mitigate issues such as anchor frame deficiencies and overlapping detection within the target region.

In the tabular data processing domain, the ETED was introduced as a prognostic classification framework for predicting seven-day patient survival outcomes. The proposed adaptive gated weighting adjustment mechanism dynamically modulates sequence information weights, effectively capturing critical temporal details and long-range dependencies. This architectural innovation enhanced the capability of the model to process high-dimensional sequential features. Comparative validation experiments demonstrated that the developed model outperformed state-of-the-art classification algorithms in terms of prediction metrics. A multimodal dataset was constructed by integrating the facial biometric features and clinical physiological parameters, leveraging an augmented perception-based prediction framework, a hybrid classification model was formulated to estimate seven-day survival probabilities. The implementation of ETED on this multimodal dataset yielded clinically actionable insights into patient prognosis. Key features including chronic health evaluation scores, MAP, GCS, FIB, and oculomotor flexibility, were significantly associated with survival outcomes. Elevated chronic health evaluation scores and MAP values correlated with improved survival likelihood, whereas higher body temperature, GCS scores, and FIB concentrations demonstrated negative prognostic relationships. These findings validate that the multimodal dataset combining facial biometrics and clinical indicators provides substantial improvements in predicting seven-day patient survival status compared with unimodal approaches.

This investigation is inherently constrained by several methodological limitations. First, the restricted selection of facial landmark regions during facial feature extraction may compromise the predictive validity and representativeness of the facial biometric data in survival outcome modeling. Second, the limited sample size of clinical cases used to train the tabular data-based prognostic classifier may impede its generalizability and predictive precision across heterogeneous patient populations with diverse pathological conditions. To mitigate these constraints, future research should prioritize expanding the facial feature dataset by incorporating additional anatomically significant regions and increasing sample diversity through the inclusion of patients from varied clinical backgrounds. These enhancements would strengthen the robustness and translational utility of the prognostic models in real-world clinical settings.

## Conclusions

In this study, a multimodal survival prediction framework was developed for clinical populations by integrating computer vision-based facial feature detection with temporal tabular data classification to enhance the prognostic accuracy of smart healthcare systems. For facial feature extraction, the proposed lightweight face-region convolutional neural network, FRegNet, incorporates a residual stem, Rstem, to deepen feature representation and mitigate information loss, along with FPAFPN, which implements adaptive scaling for precise multi resolution fusion. Comparative experiments validated its superiority over state-of-the-art algorithms, achieving AP of 0.922, AR of 0.933, mAP of 0.987, and precision of 0.98–significantly outperforming mask R-CNN variants (e.g., mask RCNN-ResNeXt with AP of 0.789 AP and mAP of 0.957), and enabling the robust detection of critical facial markers, such as scleral jaundice, reflecting disease progression. For temporal modeling, the ETED framework integrates adaptive attention and gated weighting mechanisms to dynamically adjust feature importance based on clinical events, outperforming transformer-based and traditional models. Specifically, the ETED variant with facial features ETEncoding-Decoding-Face, achieves an accuracy of 0.916, precision of 0.850, recall of 0.895, F1-score of 0.884, and AUC of 0.947, surpassing gradient boosting, with an accuracy of 0.922, but AUC of 0.669 in comprehensive reliability. By integrating FRegNet-extracted facial features with longitudinal clinical indicators, the framework constructs a seven-day survival prediction model that outperforms unimodal approaches, validating the value of synergizing systemic physiological status and facial disease manifestations. Correlation analysis revealed chronic health evaluation scores and MAP are positively correlated with survival, whereas temperature, GCS scores, and FIB levels are negatively correlated, offering actionable clinical insights. Collectively, this study contributes three key advancements: FRegNet sets a new benchmark in clinical facial detection, ETED enables context-aware modeling of irregular clinical data, and multimodal integration improves survival prediction accuracy, providing a novel paradigm for smart healthcare and supporting precise personalized preventive strategies.

## Data Availability

The raw data supporting the conclusions of this article will be made available by the authors, without undue reservation.

## Appendix

**Appendix A:** Clinical dataset indicators

Indicator	Description
Sex	Refers to the sex of the individual
Apache II	Scoring system for assessing illness severity and predicting mortality
Temperature	Body temperature
Systolic pressure	Systolic blood pressure
Diastolic pressure	Diastolic blood pressure
MAP	Mean arterial pressure
Heart rate	Heart rate
Respiratory rate	Respiratory rate
Partial pressure of oxygen	Partial pressure of oxygen in the blood
pH	Blood pH
Na	Sodium concentration in the blood
K	Potassium concentration in the blood
Cr	Creatinine level
HCT	Red blood cell pressure
WBC	White blood cell count
GCS	Glasgow coma score
AE score	Acute physiological and chronic health evaluation score
Chronic health evaluation	Evaluation of chronic health conditions
Hemoglobin	Hemoglobin level
Anemia	Presence of anemia
Platelet	Platelet count
PCO_2_	Partial pressure of carbon dioxide

## Abbreviations

AP: Average precision
mAP: Mean average precision
RCNN: Region-based convolutional neural networks
ETED: Enhanced temporal encoding-decoding
AUC: Area under the curve
AR: Average recall
IoU: Intersection-over-union
FPAFPN: Face pyramid aggregation feature pyramid network
ETED: Enhanced temporal encoding-decoding
LSTM: Long short-term memory
GCS: Glasgow Coma Scale

## References

[1] Li ZS, Zhang H, Baraghtha S, Mu JB, Matniyaz Y, Jiang XY et al (2022) Short- and mid-term survival prediction in patients with acute type A aortic dissection undergoing surgical repair: based on the systemic immune-inflammation index. J Inflamm Res 15:5785–5799. 10.2147/JIR.S382573 10.2147/JIR.S382573 PMC9553311 36238764

[2] Latenstein AEJ, van Roessel S, van der Geest LGM, Bonsing BA, Dejong CHC, Groot Koerkamp B et al (2020) Conditional survival after resection for pancreatic cancer: a population-based study and prediction model. Ann Surg Oncol 27(7):2516–2524. 10.1245/s10434-020-08235-w 10.1245/s10434-020-08235-w PMC7311496 32052299

[3] Raji CG, Safna AK (2022) Computational methods for predicting the outcome of thoracic transplantation. J Big Data 9(1):58. 10.1186/s40537-022-00609-z

[4] Sarolidou G, Axelsson J, Sundelin T, Lasselin J, Regenbogen C, Sorjonen K et al (2019) Emotional expressions of the sick face. Brain Behav Immun 80:286–291. 10.1016/j.bbi.2019.04.003 10.1016/j.bbi.2019.04.003 30953768

[5] Utkin LV, Zaborovsky VS, Kovalev MS, Konstantinov AV, Politaeva NA, Lukashin AA (2021) Uncertainty interpretation of the machine learning survival model predictions. IEEE Access 9:120158–120175. 10.1109/ACCESS.2021.3108341

[6] Kuehne M, Polotzek L, Haghikia A, Zaehle T, Lobmaier JS (2023) I spy with my little eye: the detection of changes in emotional faces and the influence of facial feedback in Parkinson disease. Eur J Neurol 30(3):622–630. 10.1111/ene.15647 10.1111/ene.15647 36435983

[7] Meng T, Guo XP, Lian W, Deng K, Gao L, Wang ZH et al (2020) Identifying facial features and predicting patients of acromegaly using three-dimensional imaging techniques and machine learning. Front Endocrinol (Lausanne) 11:492. 10.3389/fendo.2020.00492 10.3389/fendo.2020.00492 PMC7403213 32849283

[8] Gao PC, Lu K, Xue J, Lyu JY, Shao L (2023) A facial landmark detection method based on deep knowledge transfer. IEEE Trans Neural Netw Learn Syst 34(3):1342–1353. 10.1109/TNNLS.2021.3105247 10.1109/TNNLS.2021.3105247 34449395

[9] Barua PD, Baygin N, Dogan S, Baygin M, Arunkumar N, Fujita H et al (2022) Automated detection of pain levels using deep feature extraction from shutter blinds-based dynamic-sized horizontal patches with facial images. Sci Rep 12(1):17297. 10.1038/s41598-022-21380-4 10.1038/s41598-022-21380-4 PMC9568538 36241674

[10] Leung TS, Maylott SE, Zeng GY, Nascimben DN, Jakobsen KV, Simpson EA (2023) Behavioral and physiological sensitivity to natural sick faces. Brain Behav Immun 110:195–211. 10.1016/j.bbi.2023.03.007 10.1016/j.bbi.2023.03.007 36893923

[11] Zhang Q, Zhou JH, Zhang B, Wu EH (2021) DSNet: dual stack network for detecting diabetes mellitus and chronic kidney disease. Inf Sci 547:945–962. 10.1016/j.ins.2020.08.074

[12] Wang Y, Ye Y, Shi S, Mao K, Zheng H, Chen X et al (2024) Prediagnosis recognition of acute ischemic stroke by artificial intelligence from facial images. Aging Cell 23(8):e14196. 10.1111/acel.14196 10.1111/acel.14196 PMC11320352 38845183

[13] Zhang Q, Wen J, Zhou JH, Zhang B (2022) Missing-view completion for fatty liver disease detection. Comput Biol Med 150:106097. 10.1016/j.compbiomed.2022.106097 10.1016/j.compbiomed.2022.106097 36244304

[14] Wu WX, Yan J, Zhao YS, Sun QC, Zhang HL, Cheng JL et al (2023) Multi-task learning for concurrent survival prediction and semi-supervised segmentation of gliomas in brain MRI. Displays 78:102402. 10.1016/j.displa.2023.102402

[15] Sun T, Wei Y, Chen W, Ding Y (2020) Genome-wide association study-based deep learning for survival prediction. Stat Med 39(30):4605–4620. 10.1002/sim.8743 10.1002/sim.8743 PMC8056253 32974946

[16] Fatima A, Shafi I, Afzal H, Mahmood K, de la Torre Díez I, Lipari V et al (2023) Deep learning-based multiclass instance segmentation for dental lesion detection. Healthcare (Basel) 11(3):347. 10.3390/healthcare11030347 10.3390/healthcare11030347 PMC9914729 36766922

[17] Wang YY, Wang DJ, Ye X, Wang YZ, Yin YQ, Jin YC (2019) A tree ensemble-based two-stage model for advanced-stage colorectal cancer survival prediction. Inf Sci 474:106–124. 10.1016/j.ins.2018.09.046

[18] Jin B, Cruz L, Gonçalves N (2020) Deep facial diagnosis: deep transfer learning from face recognition to facial diagnosis. IEEE Access 8:123649–123661. 10.1109/ACCESS.2020.3005687

[19] Zhao XT, Xu TK, Peng L, Li SY, Zhao YM, Liu HW et al (2023) Recognition and segmentation of teeth and mandibular nerve canals in panoramic dental X-rays by Mask RCNN. Displays 78:102447. 10.1016/j.displa.2023.102447

[20] Hoorali F, Khosravi H, Moradi B (2023) An automatic method for microscopic diagnosis of diseases based on URCNN. Biomed Signal Process Control 80:104240. 10.1016/j.bspc.2022.104240

[21] Indumathi V, Siva R (2023) An efficient lung disease classification from X-ray images using hybrid Mask-RCNN and BiDLSTM. Biomed Signal Process Control 81:104340. 10.1016/j.bspc.2022.104340

[22] Fan M, Zheng HZ, Zheng S, You C, Gu YJ, Gao X et al (2020) Mass detection and segmentation in digital breast tomosynthesis using 3D-Mask region-based convolutional neural network: a comparative analysis. Front Mol Biosci 7:599333. 10.3389/fmolb.2020.599333 10.3389/fmolb.2020.599333 PMC7686533 33263004

[23] Moccia S, Fiorentino MC, Frontoni E (2021) Mask-R2 CNN: a distance-field regression version of Mask-RCNN for fetal-head delineation in ultrasound images. Int J Comput Assist Radiol Surg 16(10):1711–1718. 10.1007/s11548-021-02430-0 10.1007/s11548-021-02430-0 PMC8580944 34156608

[24] Mitate E, Inoue K, Sato R, Shimomoto Y, Ohba S, Ogata K et al (2022) Application of the sliding window method and Mask-RCNN method to nuclear recognition in oral cytology. Diagn Pathol 17, 62. 10.1186/s13000-022-01245-0 10.1186/s13000-022-01245-0 PMC9344779 35918750

[25] Evain E, Raynaud C, Ciofolo-Veit C, Popoff A, Caramella T, Kbaier P et al (2021) Breast nodule classification with two-dimensional ultrasound using Mask-RCNN ensemble aggregation. Diagn Interv Imaging 102(11):653–658. 10.1016/j.diii.2021.09.002 10.1016/j.diii.2021.09.002 34600861

[26] Long K, Tang L, Pu XR, Ren YZ, Zheng MX, Gao L et al (2021) Probability-based Mask R-CNN for pulmonary embolism detection. Neurocomputing 422:345–353. 10.1016/j.neucom.2020.10.022

[27] Hussain E, Mahanta LB, Das CR, Choudhury M, Chowdhury M (2020) A shape context fully convolutional neural network for segmentation and classification of cervical nuclei in Pap smear images. Artif Intell Med 107:101897. 10.1016/j.artmed.2020.101897 10.1016/j.artmed.2020.101897 32828445

[28] Vieira PM, Freitas NR, Lima VB, Costa D, Rolanda C, Lima CS (2021) Multi-pathology detection and lesion localization in WCE videos by using the instance segmentation approach. Artif Intell Med 119:102141. 10.1016/j.artmed.2021.102141 10.1016/j.artmed.2021.102141 34531016

[29] Felfeliyan B, Hareendranathan A, Kuntze G, Jaremko JL, Ronsky JL (2022) Improved-Mask R-CNN: towards an accurate generic MSK MRI instance segmentation platform (data from the Osteoarthritis Initiative). Comput Med Imaging Graph 97:102056. 10.1016/j.compmedimag.2022.102056 10.1016/j.compmedimag.2022.102056 35364383

[30] Masood M, Nazir T, Nawaz M, Mehmood A, Rashid J, Kwon HY et al (2021) A novel deep learning method for recognition and classification of brain tumors from MRI images. Diagnostics (Basel) 11(5):744. 10.3390/diagnostics11050744 10.3390/diagnostics11050744 PMC8143310 33919358

[31] Jin J, Zhang QQ, Dong B, Ma T, Mei XC, Wang X et al (2022) Automatic detection of early gastric cancer in endoscopy based on Mask region-based convolutional neural networks (Mask R-CNN) (with video). Front Oncol 12:927868. 10.3389/fonc.2022.927868 10.3389/fonc.2022.927868 PMC9630732 36338757

[32] Lin S, Li ZG, Fu BW, Chen SP, Li X, Wang Y et al (2020) Feasibility of using deep learning to detect coronary artery disease based on facial photo. Eur Heart J 41(46):4400–4411. 10.1093/eurheartj/ehaa640 10.1093/eurheartj/ehaa640 32818267

[33] Zhuang Y, McDonald M, Uribe O, Yin XW, Parikh D, Southerland AM et al (2020) Facial weakness analysis and quantification of static images. IEEE J Biomed Health Inform 24(8):2260–2267. 10.1109/JBHI.2020.2964520 10.1109/JBHI.2020.2964520 31944968

[34] Wu HJ, Gao R, Sheng YP, Chen B, Li S (2020) SDAE-GAN: enable high-dimensional pathological images in liver cancer survival prediction with a policy gradient based data augmentation method. Med Image Anal 62:101640. 10.1016/j.media.2020.101640 10.1016/j.media.2020.101640 32120270

[35] Wang CY, Guo JL, Zhao N, Liu Y, Liu XY, Liu GJ et al (2020) A cancer survival prediction method based on graph convolutional network. IEEE Trans NanoBiosci 19(1):117–126. 10.1109/TNB.2019.2936398 10.1109/TNB.2019.2936398 31443039

[36] Chen ZB, Wei QF (2022) Developing an improved survival prediction model for disease prognosis. Biomolecules 12(12):1751. 10.3390/biom12121751 10.3390/biom12121751 PMC9775036 36551179

[37] Ma BS, Yan G, Chai BJ, Hou XY (2022) XGBLC: an improved survival prediction model based on XGBoost. Bioinformatics 38(2):410–418. 10.1093/bioinformatics/btab675 10.1093/bioinformatics/btab675 34586380

[38] Li ZY, Wu XL, Gao XY, Shan F, Ying XJ, Zhang Y et al (2020) Development and validation of an artificial neural network prognostic model after gastrectomy for gastric carcinoma: an international multicenter cohort study. Cancer Med 9(17):6205–6215. 10.1002/cam4.3245 10.1002/cam4.3245 PMC7476835 32666682

[39] Lee CJ, Baek B, Cho SH, Jang TY, Jeon SE, Lee S et al (2023) Machine learning with in silico analysis markedly improves survival prediction modeling in colon cancer patients. Cancer Med 12(6):7603–7615. 10.1002/cam4.5420 10.1002/cam4.5420 PMC10067044 36345155

[40] Pongnikorn D, Phinyo P, Patumanond J, Daoprasert K, Phothong P, Siribumrungwong B (2021) Individualized prediction of breast cancer survival using flexible parametric survival modeling: analysis of a hospital-based national clinical cancer registry. Cancers (Basel) 13(7):1567. 10.3390/cancers13071567 10.3390/cancers13071567 PMC8037061 33805407

[41] Atallah DM, Badawy M, El-Sayed A, Ghoneim MA (2019) Predicting kidney transplantation outcome based on hybrid feature selection and KNN classifier. Multimed Tools Appl 78(14):20383-20407. 10.1007/s11042-019-7370-5

[42] Ding DY, Lang TY, Zou DL, Tan JW, Chen J, Zhou L et al (2021) Machine learning-based prediction of survival prognosis in cervical cancer. BMC Bioinformatics 22(1):331. 10.1186/s12859-021-04261-x 10.1186/s12859-021-04261-x PMC8207793 34134623

[43] Hao YR, Jing XY, Sun QX (2022) Joint learning sample similarity and correlation representation for cancer survival prediction. BMC Bioinformatics 23(1):553. 10.1186/s12859-022-05110-1 10.1186/s12859-022-05110-1 PMC9761951 36536289

[44] Wiltgen T, Fleischmann DF, Kaiser L, Holzgreve A, Corradini S, Landry G et al (2022) 18F-FET PET radiomics-based survival prediction in glioblastoma patients receiving radio(chemo)therapy. Radiat Oncol 17(1):198. 10.1186/s13014-022-02164-6 10.1186/s13014-022-02164-6 PMC9719240 36461120

